# Ought the Formation of Dental Schools Be Limited?

**Published:** 1895-11

**Authors:** C. W. Stainton

**Affiliations:** Buffalo, N. Y.


### Ought the Formation of Dental Schools be Limited?

BY C. W. STAINTON, D.D.S., BUFFALO, N. Y.

Abstract of paper read at American Dental Association, Aug., 1895.

In the course of his remarks on this important subject Dr. Stainton stated that the number of physicians practicing in 1895 is about 120,000, an average of one for every 587 of population; that medical schools in 1895 numbered 156. Number of dentists in the United States in 1895 about 25,000, or one for every 3,134 of population. (He gave numerous comparative tables which we cannot here append for want of space—Ed.) Number of dental schools, 1895, is 48.

Continuing, he said: "What can we deduce from these estimates? That the dental ranks are as full to-day in the United States, in proportion to the demands, as the medical ranks. The over-crowding of our ranks in any locality is prolific in cheap and nasty practitioners, and the lowering of the standard and character of our specialty. The over-production of dentists is not a good thing, either for us or the public." He said we could restrain this somewhat by raising the standard of admission to our dental colleges and lengthening the course of study. "But," he continued, "the multiplication of dental colleges is the chief danger in this direction. . . . . Dental schools are not being formed now from any need felt for them as an educational necessity. Two impulses control this matter:

*First.* Personal ambition to have a position in, and be connected with, a dental college, for the prominence it is supposed to give.

*Second.* A purely commercial spirit on the part of medical schools to have a dental department. Already over 60 percent. of our schools are appendages to medical schools, and medical influences and elements are fostering, more than any and all other influences and elements, the formation of new dental schools.

Regarding the remedy for the formation of new colleges, he said the Faculty Association should serve notice that hereafter no dental school would be accepted under any circumstances unless consent of the Association was first asked and received for its formation. The moral force of 25,000 dentists, yea, of the world, would sustain it in such a movement.

